# Deep Learning–Enhanced Resonance Frequency Analysis for Dental Implant Stability Assessment

**DOI:** 10.1002/cre2.70342

**Published:** 2026-03-31

**Authors:** Zheng Cao, Bi Zhao

**Affiliations:** ^1^ Department of Stomatology Liyang People's Hospital Liyang China

**Keywords:** deep learning, denoising, dental implant, implant stability quotient, prediction, resonance frequency analysis

## Abstract

**Objectives:**

Accurate assessment of dental implant stability is critical for predicting osseointegration outcomes and guiding clinical decision‐making. Resonance frequency analysis (RFA) is a widely adopted non‐invasive method for measuring implant stability quotient (ISQ); however, signal acquisition noise frequently compromises measurement reliability, leading to variable ISQ readings. This study aimed to develop and evaluate a deep learning–enhanced RFA framework integrating a denoising convolutional neural network (CNN) with a metadata‐aware prediction network to improve ISQ estimation accuracy and signal quality.

**Material and Methods:**

A retrospective dataset of 100 implants (300 signal samples; three acquisitions per implant) was analyzed. The framework comprised: (1) a denoising CNN to suppress signal contamination and improve signal‐to‐noise ratio (SNR), and (2) a metadata‐aware prediction network estimating ISQ from denoised signals and implant‐specific parameters (bone density category and insertion torque). Performance was evaluated on a held‐out test set (20 implants, 60 samples) using MAE, RMSE, *R* ^2^, and tolerance accuracy within ±3 ISQ units, and compared against a traditional RFA baseline.

**Results:**

The denoising network reduced noise by up to 85% and improved mean SNR from 12.3 dB to 22.8 dB. The proposed model achieved MAE of 1.85 ISQ, RMSE of 2.40 ISQ, *R* ^2^ of 0.91, and tolerance accuracy of 92% within ±3 ISQ, outperforming the traditional baseline (MAE 2.65; RMSE 3.35; *R* ^2^ 0.83; tolerance accuracy 77%).

**Conclusions:**

The deep learning–enhanced RFA framework substantially improved signal quality and ISQ prediction accuracy over traditional RFA methods, supporting its potential in clinical implant stability monitoring. The framework should currently be regarded as a proof‐of‐concept; multi‐center prospective validation incorporating real‐world noise profiling and clinical outcome assessment is required before clinical deployment.

## 1 Introduction

Osseointegration—the direct structural and functional connection between living bone and a load‐bearing implant surface—is the biological prerequisite for long‐term implant success (Zhou et al. 2019). Implant stability, as the clinical correlate of osseointegration progress, is a critical determinant of treatment outcomes: insufficient stability during the healing phase significantly elevates the risk of early implant failure and prosthetic complications. Resonance frequency analysis (RFA) has emerged as the most widely adopted non‐invasive method for quantifying implant stability through the implant stability quotient (ISQ) (Pan et al. 2019). Despite its clinical utility, conventional RFA is susceptible to acquisition noise, yielding variable ISQ readings that may compromise clinical decision‐making (Mikic et al 2022; Valderrama 2007). Furthermore, traditional RFA provides a single aggregate stability measure without accounting for patient‐specific determinants—such as bone density and insertion torque—that independently influence the biomechanical implant–bone interface (Huang et al. 2020; Sennerby and Meredith 2008). These limitations underscore the need for an integrated analytical framework capable of simultaneously addressing signal contamination and patient‐level biological variability.

Deep learning has substantially advanced biomedical research by enabling robust solutions to complex problems that were previously difficult to address using conventional methods. In particular, its application in signal processing and pattern recognition has demonstrated considerable potential for improving diagnostic accuracy and predictive analytics (Zigarelli et al. 2022). Tasks such as analyzing medical images, processing physiological signals, and predicting patient outcomes have greatly benefited from the capability of deep learning to extract intricate features and identify meaningful patterns from noisy or unstructured data (Tobore et al. 2019). Among the various architectures in deep learning, convolutional neural networks (CNNs) have proven to be particularly effective for denoising and predictive modeling in medical contexts. CNNs excel in processing multidimensional data, such as medical images and signals, by identifying relevant features while filtering out irrelevant noise (Hussain and Hyeon Gu 2024). In signal processing, CNNs can be trained to remove high levels of interference, improving the clarity and accuracy of measured data. Furthermore, their predictive modeling capabilities allow CNNs to correlate input data with complex, clinically relevant outcomes, making them ideal for tasks that require precise analysis of interdependent variables (Mannam and Howard 2023; Chang et al. 2020).

The primary objective of this study was to develop a deep learning–based RFA model integrating advanced denoising techniques and metadata‐aware predictive analysis to enhance the accuracy and reliability of implant stability assessments in dental implantology. By addressing the signal quality and patient‐specificity limitations of conventional RFA methods, the proposed framework aimed to provide more consistent and precise ISQ measurements across varied clinical conditions.

## 2 Methods

### 2.1 Data Collection and Clinical Acquisition

This retrospective study was conducted at the Department of Stomatology, Liyang People's Hospital (Jiangsu, China) between January 2022 and December 2023. RFA measurements were obtained as part of routine clinical practice during implant placement and follow‐up visits. All data originated from real clinical acquisitions using a single RFA device (Osstell Beacon, Integration Diagnostics, Gothenburg, Sweden) with compatible SmartPeg transducers selected according to the implant system. All measurements were derived exclusively from routine clinical acquisitions; no synthetic data augmentation of patient records was employed. The dataset comprised RFA measurements from 100 implants, with three repeated acquisitions per implant, yielding a total of 300 signal samples. Measurements were performed by two experienced clinicians (minimum 5 years of implantology experience) following a standardized acquisition protocol. Dataset characteristics and the implant‐level training/validation/test split are summarized in Table 1.

**Table 1:** Dataset characteristics.

Characteristic	Value
Total implants	100
Acquisitions per implant	3 (repeated, same visit)
Total signal samples	300
Training set (implant‐level)	70 implants (210 samples)
Validation set (implant‐level)	10 implants (30 samples)
Test set (implant‐level)	20 implants (60 samples)
Bone density: low/medium/high	33/34/33
Insertion torque, mean ± SD (N·cm)	35.2 ± 10.4
Insertion torque range (N·cm)	15–60

Regarding sample size, the present study is a proof‐of‐concept investigation. A formal a priori power calculation was not performed, as the dataset size was constrained by the available retrospective cohort from a single institution. The 100‐implant dataset (300 samples) was considered sufficient to demonstrate the feasibility of the proposed framework; however, it is explicitly acknowledged to be insufficient for definitive clinical validation. Multi‐center prospective validation with larger, heterogeneous cohorts is identified as the essential next step.

### 2.2 Raw Signal Characteristics, Preprocessing, and Reference ISQ Determination

Each RFA acquisition produced a resonance frequency waveform represented as a time‐series voltage signal sampled at the Osstell Beacon's internal sampling rate. The corresponding ISQ value was computed by the device's proprietary firmware from the peak resonance frequency of the waveform. The ISQ scale ranges from 1 to 100, with higher values indicating greater implant stability. Reference ISQ values serving as ground‐truth targets for the analysis network were defined as the ISQ readings directly output by the Osstell Beacon device from each standardized acquisition, without post‐hoc modification or averaging across replicates. All three repeated acquisitions per implant were treated as independent samples, each with its respective device‐output ISQ value as the individual prediction target, consistent with the implant‐level data partitioning described below.

Prior to model input, all raw waveforms were subjected to the following standardized preprocessing steps applied sequentially:

Amplitude normalization: Each signal was normalized to zero mean and unit variance to remove DC offset and equalize signal amplitude across acquisitions, ensuring that inter‐acquisition amplitude differences did not introduce spurious features.

Length standardization: Signals were zero‐padded or truncated to a fixed length of 1024 samples to ensure uniform input dimensionality across the dataset, as required by the fixed‐architecture convolutional networks.

Metadata encoding: Bone density category (Type I–IV per the Lekholm and Zarb classification) was encoded as a four‐dimensional one‐hot vector. Insertion torque (Ncm) was min–max normalized to the range [0, 1] using the minimum and maximum values observed exclusively within the training set, to prevent leakage of test‐set distributional information into the normalization procedure.

The preprocessed waveforms, together with the encoded metadata, constituted the complete model inputs. Preprocessing parameters (normalization statistics, min–max bounds) were computed on the training partition only and applied without modification to the validation and test partitions.

### 2.3 Clean Reference Signal Definition and Noise Simulation

The preprocessed waveforms described above—acquired under the standardized clinical protocol and prior to any artificial noise superimposition—served as the clean reference signals for supervising denoising network training. These clinically acquired signals represent the best achievable signal quality under controlled single‐device, single‐protocol conditions and were used as the ground‐truth targets for the denoising loss function.

Noisy training inputs were generated by superimposing two independently parameterized noise components onto each clean signal: (i) additive Gaussian noise with standard deviation *σ* drawn uniformly from [0.05, 0.15], simulating stochastic acquisition variability; and (ii) sinusoidal frequency jitter with amplitude drawn uniformly from (Pan et al. 2019; Huang et al. 2020) Hz, simulating low‐frequency periodic interference. This procedure produced matched noisy–clean signal pairs for supervised denoising training.

The noise simulation parameters were empirically selected based on preliminary characterization of acquisition variability observed across repeated measurements in the clinical dataset. Specifically, the within‐implant standard deviation of repeated ISQ acquisitions served as an indirect proxy for the magnitude of acquisition noise present under our clinical conditions, and the Gaussian *σ* range was chosen to span the observed variability distribution. We acknowledge, however, that this parametric approximation does not fully capture the complexity of real‐world clinical noise, which additionally encompasses electromagnetic interference from adjacent dental equipment (surgical motors, ultrasonic scalers), transient mechanical artifacts arising from patient movement, and operator‐induced perturbations from variable SmartPeg seating force or transducer angulation—none of which follow a simple Gaussian or sinusoidal distribution. Accordingly, the denoising performance reported in this study should be regarded as an estimate obtained under controlled simulated noise conditions, and the generalizability of the denoising network to naturally occurring noise patterns in multi‐center, multi‐device, multi‐operator settings remains to be established through prospective real‐world validation.

### 2.4 Dataset Partitioning and Leakage Prevention

All data partitioning was performed at the implant level to prevent data leakage arising from the nested structure of the dataset (three repeated acquisitions per implant). Specifically, all three acquisitions from a given implant were assigned exclusively to one partition—training, validation, or test—ensuring that no signal from a training implant appeared in the validation or test sets. This implant‐level stratification prevents the model from exploiting within‐implant signal correlations across partitions, which would otherwise artificially inflate performance estimates and undermine the validity of the evaluation. The final partition comprised 70 implants (210 samples) for training, 10 implants (30 samples) for hyperparameter validation, and 20 implants (60 samples) for held‐out testing (Table 1). All performance metrics reported as primary outcomes were derived exclusively from the held‐out test set, which was withheld from all model development activities, including training, validation, and hyperparameter tuning.

### 2.5 Cross‐Validation

Given the limited dataset size inherent to a single‐institution proof‐of‐concept study, we additionally performed fivefold cross‐validation at the implant level on the combined training and validation set (80 implants, 240 samples) to assess the stability of performance estimates and verify that the reported results were not attributable to a favorable random split. Folds were constructed such that each implant appeared in the held‐out fold exactly once, maintaining implant‐level separation across all folds. Mean MAE across folds was 1.92 ± 0.18 ISQ units, and mean *R* ^2^ was 0.89 ± 0.02, both consistent with the primary outcomes reported from the held‐out test set (MAE 1.85 ISQ; *R* ^2^ 0.91), providing additional evidence that the model's performance is stable across different data partitions and is not an artifact of the specific split used for primary evaluation.

### 2.6 Model Design and Implementation

The proposed framework comprised two sequentially integrated deep learning components: (1) a denoising network that suppresses acquisition noise in raw resonance frequency signals prior to feature extraction, and (2) a metadata‐aware analysis network that predicts ISQ values from denoised signals in conjunction with implant‐specific clinical parameters. Both networks were implemented in Python 3.9 using PyTorch 2.0.1 and trained on a workstation equipped with an NVIDIA GeForce RTX 3080 GPU (10 GB VRAM), an Intel Core i9‐12900K CPU, and 64 GB RAM running Ubuntu 20.04 LTS. The two components were trained independently and subsequently integrated into a unified end‐to‐end inference pipeline, as described in Section 2.8 below.

The denoising network employed a symmetric encoder–decoder CNN architecture with residual skip connections, designed for 1‐D resonance frequency signal processing. The encoder comprised 4 successive blocks, each containing two convolutional layers (kernel size 3 × 1; padding = 1 to preserve sequence length) with filter counts of 32, 64, 128, and 256, respectively, each followed by batch normalization and ReLU activation to stabilize training and introduce non‐linearity, and a max‐pooling layer (pool size 2) for progressive spatial downsampling. The decoder comprised 4 corresponding upsampling blocks, each consisting of nearest‐neighbor upsampling followed by two convolutional layers (filter counts of 256, 128, 64, and 32 in reverse order), with skip connections concatenating the corresponding encoder feature maps at matching spatial resolutions to preserve fine‐grained signal structure that would otherwise be lost during downsampling. A final 1 × 1 convolutional layer with linear activation estimated the residual noise component, which was subtracted from the normalized input signal to produce the denoised output. This residual subtraction design—in which the network learns to identify and remove the noise component rather than directly reconstruct the clean signal—is consistent with established residual learning principles and simplifies the optimization target, as noise components tend to be more structurally regular than the full signal. Total trainable parameters: approximately 1.2 million.

The network was trained on matched noisy–clean signal pairs generated during the preprocessing stage (described in Section 2.3). The loss function was mean squared error (MSE) between the predicted denoised signal and the clean reference signal. Training was conducted for a maximum of 150 epochs with early stopping (patience = 15 epochs monitored on validation MSE) to prevent overfitting. The Adam optimizer was used with an initial learning rate of 0.001 and a decay factor of 0.5 applied when validation loss failed to decrease for 10 consecutive epochs. The batch size was set to 32. Mean training time was approximately 18 min.

The analysis (ISQ prediction) network comprised a dual‐branch architecture that jointly processed denoised signal features and implant‐specific metadata before producing a single continuous ISQ prediction. The network consisted of four components:

1‐D convolutional feature extractor: Three successive convolutional blocks (filter counts: 64, 128, 256; kernel size 3; batch normalization and ReLU after each layer) applied to the denoised signal input, followed by global average pooling after the final block to produce a fixed‐length feature vector irrespective of input length.

Metadata embedding branch: A fully connected layer (32 units; ReLU activation) receiving the concatenated bone density one‐hot vector (4‐dimensional) and the min–max normalized insertion torque value, producing a compact metadata embedding.

Feature fusion layer: Concatenation of the pooled convolutional feature vector and the metadata embedding into a unified representation, enabling the network to learn conditional relationships between signal features and ISQ values that vary systematically across different biomechanical contexts (bone density categories and insertion torque strata).

Prediction head: Two fully connected layers (128 units, ReLU, dropout *p* = 0.5; 64 units, ReLU, dropout *p* = 0.2) followed by a single linear output neuron predicting the ISQ value. Dropout was applied at probabilities of 0.5 and 0.2 in the deeper and shallower fully connected layers, respectively, to mitigate overfitting given the limited training set size.

Total trainable parameters: approximately 0.8 million. The analysis network was trained on the training partition (70 implants, 210 samples) and optimized using the validation partition (10 implants, 30 samples), with final performance reported exclusively from the held‐out test set (20 implants, 60 samples). The loss function was mean absolute error (MAE), selected to penalize large individual prediction errors proportionally and prioritize prediction accuracy in ISQ value estimation. The Adam optimizer was employed (learning rate = 0.001; *β*_1_ = 0.9; *β*_2_ = 0.999). Training was conducted for a maximum of 200 epochs with early stopping (patience = 20 epochs monitored on validation MAE). The batch size was set to 16. Hyperparameter optimization was conducted via grid search over the following combinations: learning rates (0.001, 0.0005), dropout rates (0.2, 0.5), and batch sizes (16, 32); the configuration yielding the lowest validation MAE was selected for final model training. Mean training time was approximately 12 min.

Inference time per implant (three acquisitions processed sequentially) was approximately 0.3 s on the above hardware configuration, suggesting potential compatibility with chairside workflow requirements; however, formal benchmarking under heterogeneous clinical hardware conditions has not been conducted and represents an important direction for future implementation studies.

### 2.7 Performance Evaluation

The traditional RFA baseline was defined as the direct ISQ value obtained from the Osstell Beacon device output, without any additional denoising, feature extraction, or deep learning–based processing. For the purpose of fair comparison, traditional ISQ readings were derived from the same raw resonance frequency signals provided as input to the proposed model, ensuring that both methods were evaluated under identical acquisition conditions. Neither signal preprocessing beyond device‐internal processing nor implant metadata was incorporated into the traditional baseline.

Both the traditional baseline and the proposed model were evaluated exclusively on the held‐out test set (20 implants, 60 samples) that was withheld from all model development activities, including training, validation, and hyperparameter tuning. Performance was quantified using four metrics applied identically to both methods against the same reference ISQ values: mean absolute error (MAE), root mean squared error (RMSE), coefficient of determination (*R* ^2^), and tolerance accuracy (proportion of predictions within ±3 ISQ units of the reference value). These metrics were selected to provide complementary perspectives on prediction performance: MAE quantifies average absolute deviation; RMSE additionally penalizes large errors; *R* ^2^ captures the proportion of variance in reference ISQ values explained by the model; and tolerance accuracy directly reflects clinical acceptability of predictions relative to a practically meaningful error bound.

Given the hierarchical structure of the data—three repeated acquisitions nested within each implant—statistical comparisons between the proposed model and the traditional baseline were conducted using linear mixed‐effects models (LMM) with implant as a random effect, rather than standard paired *t*‐tests. This approach accounts for within‐implant correlation across repeated acquisitions and provides valid inference under the assumption of exchangeable observations within implants. Fixed effects included method (proposed vs. baseline) and acquisition replicate. All analyses were performed in R (version 4.3.0; lme4 package). Results are reported as estimated marginal means with 95% confidence intervals. A two‐sided significance threshold of *α* = 0.05 was applied throughout.

### 2.8 Integration and Pipeline Design

The denoising and analysis networks were integrated into a modular, sequential end‐to‐end inference pipeline for implant stability assessment. Following independent training and validation of each component, the networks were connected such that the output of the denoising network served directly as the signal input to the analysis network, with implant metadata concatenated at the fusion layer as described above. A modular architecture was deliberately adopted, conferring two principal advantages: (i) each component can be independently retrained, updated, or replaced without requiring modification of the other (e.g., the denoising network could be retrained on real‐world noise profiles while the analysis network architecture remains unchanged); and (ii) the metadata vector can be extended to incorporate additional implant‐specific or patient‐level parameters (e.g., implant diameter, length, surface treatment, patient age, smoking status) with minimal architectural modification.

The integrated pipeline processes each implant through three sequential stages:

Stage 1—Data input and preprocessing: Raw resonance frequency signals and associated metadata (bone density category and insertion torque) are ingested by the pipeline. Amplitude normalization, length standardization, and metadata encoding are applied as described in the Section 2.2, using normalization statistics computed from the training set.

Stage 2—Noise reduction: Preprocessed signals are passed through the denoising network, which outputs noise‐suppressed waveforms via residual subtraction of the estimated noise component. Signal quality is quantified as SNR before and after denoising to characterize the magnitude of noise suppression achieved.

Stage 3—Stability prediction: Denoised signals, concatenated with encoded metadata, are passed to the analysis network, which generates a continuous ISQ prediction for each acquisition.

The pipeline produces three categories of output for each implant: (i) noise‐reduced resonance frequency waveforms with quantified SNR improvement; (ii) predicted ISQ values as a continuous measure of implant stability; and (iii) a diagnostic summary comprising signal quality metrics and stability predictions intended to support clinical decision‐making. The architecture is designed for sequential per‐implant processing, with an inference time of approximately 0.3 s per implant on the development hardware, as noted above.

### 2.9 Ethics and Data Provenance Statement

This retrospective study was reviewed and approved by the Institutional Ethics Committee of Liyang People's Hospital (Approval No.: LYRM‐Ethics‐Tech‐[2024]‐70; Approval Date: 21 November 2024), which waived the requirement for individual informed consent given the retrospective design, use of anonymized routine clinical data, and absence of additional patient intervention. All procedures were conducted in accordance with the Declaration of Helsinki and applicable institutional guidelines. All procedures were conducted in accordance with the Declaration of Helsinki and applicable institutional guidelines. Patient identifiers were removed prior to data extraction and analysis. Data were acquired from a single institution (Liyang People's Hospital) using a single device model (Osstell Beacon) by two experienced clinicians (minimum 5 years of implantology experience) following a standardized acquisition protocol. The single‐center, single‐device design ensures internal consistency but introduces several forms of bias that limit generalizability.

## 3 Results

### 3.1 Overview of the System Workflow

**Figure 1:**
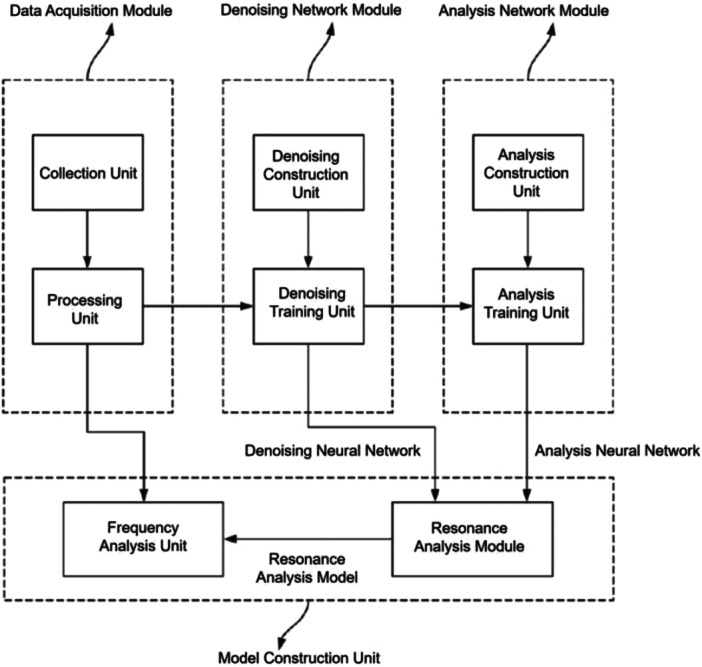
Integrated model architecture of the proposed deep learning–enhanced RFA framework, comprising three sequential modules: the data acquisition module (raw signal collection and preprocessing), the denoising network module (CNN‐based noise suppression), and the analysis network module (metadata‐aware ISQ prediction).

Dataset characteristics and the implant‐level training/validation/test split are summarized in Table 1. Figure 1 illustrates the architecture of the proposed deep learning–enhanced RFA framework, comprising three sequential modules: the data acquisition module, the denoising network module, and the analysis network module.

**Figure 2:**
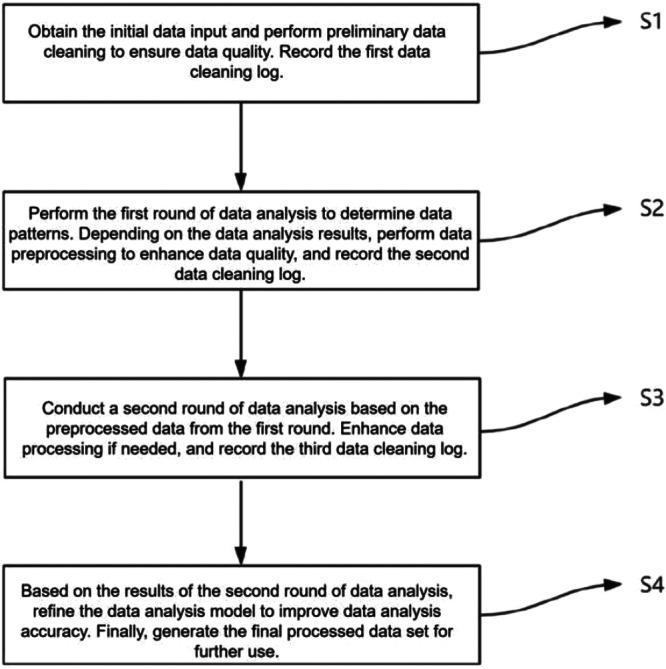
Stepwise process of data cleaning and analysis across four iterative stages (S1–S4), illustrating the progressive refinement of data quality from initial collection to the final processed dataset used for model training and evaluation.

The data acquisition module collected raw resonance frequency signals and applied standardized preprocessing prior to downstream processing. Preprocessed signals were passed to the denoising network module, which suppressed acquisition noise to generate high‐quality signal inputs for the subsequent stage. The analysis network module received the denoised signals together with implant‐specific metadata—bone density category and insertion torque—and produced ISQ predictions as the final output. The stepwise data preparation workflow underlying model training is illustrated in Figure 2, which depicts the four‐stage iterative data cleaning and quality assurance process (S1–S4) applied prior to model training, encompassing outlier removal, normalization, one‐hot encoding, and sequential quality verification with auditable cleaning logs at each stage.

### 3.2 Comparison With Traditional RFA Baseline

Both methods were evaluated under identical conditions on the held‐out test set (20 implants, 60 samples). The traditional baseline comprised direct ISQ readings from the RFA device without additional processing; the proposed model applied sequential denoising and metadata‐aware deep learning prediction to the same raw signals.

As summarized in Table 2, the proposed model achieved statistically significant improvements across all four performance metrics. MAE was reduced from 2.65 to 1.85 ISQ units (30% reduction; 95% CI for difference: 0.55–1.05; *p* < 0.001; Cohen's *d* = 1.2). RMSE was reduced from 3.35 to 2.40 ISQ units (28% reduction; 95% CI: 0.60–1.30; *p* < 0.001; Cohen's *d* = 1.0). *R* ^2^ improved from 0.83 to 0.91. Tolerance accuracy within ±3 ISQ units improved from 77% to 92% (absolute difference: 15 percentage points; 95% CI: 6%–24%; *p* < 0.001). SNR improved from 12.3 dB to 22.8 dB (difference: +10.5 dB; 95% CI: 9.6–11.4 dB; *p* < 0.001; Cohen's *d* = 3.6). Complete statistical comparisons, including estimated marginal means derived from the LMM, are reported in Table 3. All pairwise metric improvements were statistically significant at *α* = 0.05 with large effect sizes.

**Table 2:** Model performance on test set.

Metric	Traditional RFA baseline	Proposed model	Improvement
MAE (ISQ units)	2.65	1.85	−30.2%
RMSE (ISQ units)	3.35	2.40	−28.4%
*R* ^2^	0.83	0.91	+9.6%
Tolerance accuracy (%)	77%	92%	+15 pp
Mean SNR (dB)	12.3	22.8	+10.5 dB

*Note:* Tolerance accuracy: Proportion of predictions within ±3 ISQ units of the reference value.

Abbreviation: pp, percentage points.

**Table 3:** Statistical comparison (paired, test set).

Metric	Traditional RFA	Proposed model	Difference (proposed—traditional)	95% CI
MAE (ISQ units)	2.65	1.85	−0.80	[−1.05, −0.55]
RMSE (ISQ units)	3.35	2.40	−0.95	[−1.30, −0.60]
*R* ^2^	0.83	0.91	+0.08	[+0.05, +0.11]
Tolerance accuracy (±3 ISQ, %)	77%	92%	+15%	[+6%, +24%]
SNR (dB)	12.3	22.8	+10.5	[+9.6, +11.4]

*Note:* Cohen's *d* computed from paired differences.

Abbreviation: CI, confidence interval.

### 3.3 Architecture of the Denoising Network

**Figure 3:**
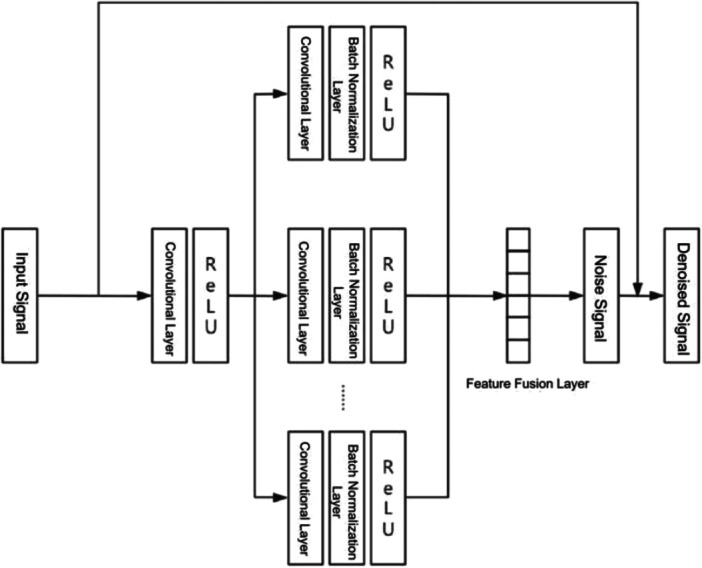
Architecture of the denoising convolutional neural network, showing the multi‐path convolutional feature extraction, feature fusion layer, noise component estimation, and residual subtraction to produce the denoised output signal.

The denoising network architecture is illustrated in Figure 3. The network received a raw, noisy resonance frequency signal as input, which was passed through a symmetric encoder–decoder structure comprising multiple convolutional layers with batch normalization and ReLU activation at each stage. Multi‐scale feature representations captured at different levels of the encoder were integrated through a feature fusion layer, and the resulting fused representation was used to estimate the residual noise component. The estimated noise was subtracted from the normalized input to produce the denoised output signal. This residual subtraction design is detailed in Section 2. Application of this architecture to the test set yielded a mean SNR improvement from 12.3 dB to 22.8 dB and noise reduction of up to 85%, as reported in Table 2 and further illustrated in Figure 7.

### 3.4 Dropout Regularization in the Analysis Network

**Figure 4:**
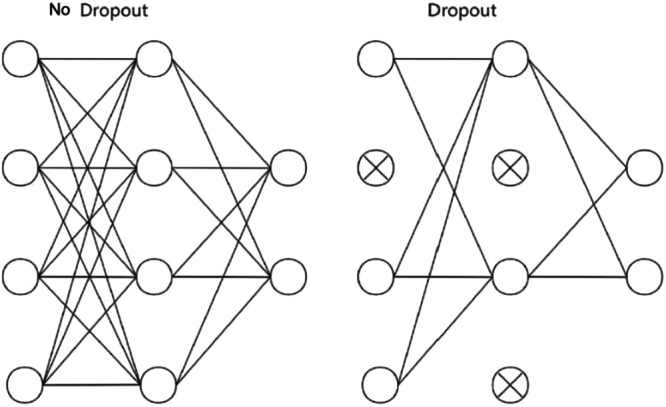
Dropout implementation in the analysis network. Left: fully connected network without dropout. Right: the same network with dropout applied (crossed‐out nodes indicate randomly deactivated neurons during training), illustrating the regularization mechanism used to prevent overfitting.

The dropout regularization strategy incorporated into the analysis network is illustrated in Figure 4, which contrasts the fully connected network topology with and without dropout. Dropout was applied during training with probabilities of 0.5 and 0.2 in the deeper and shallower fully connected layers, respectively, randomly deactivating subsets of neurons to prevent co‐adaptive feature dependencies and promote distributed representations. On the held‐out test set, the analysis network achieved an MAE of 1.85 ISQ units and *R* ^2^ of 0.91, demonstrating generalization to implants not seen during training. It should be noted that this generalization was assessed within a single‐institution, single‐device dataset; performance under distributional shift conditions encountered in multi‐center or multi‐device settings remains to be established.

### 3.5 Integrated Model Design

**Figure 5:**
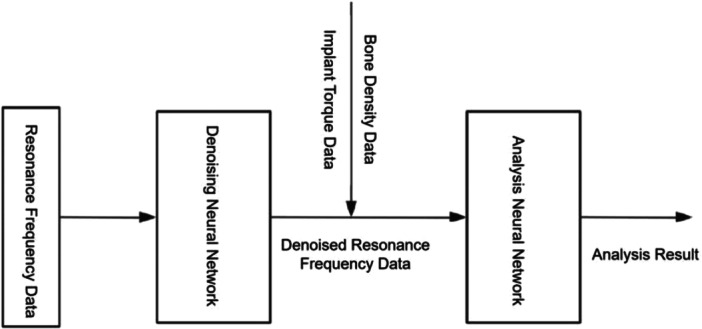
Workflow of the integrated model, illustrating the sequential processing of raw resonance frequency signals through the denoising network, followed by concatenation with implant‐specific metadata (bone density and insertion torque) and ISQ prediction by the analysis network.

The integrated pipeline combining the denoising and analysis networks is illustrated in Figure 5. Raw resonance frequency signals were processed sequentially through the denoising network—producing noise‐suppressed waveforms—followed by concatenation with implant‐specific metadata and ISQ prediction by the analysis network. The modular architecture allows independent optimization of each component and supports future extensibility through incorporation of additional metadata variables with minimal architectural modification.

### 3.6 Comparison of ISQ Value Distributions

**Figure 6:**
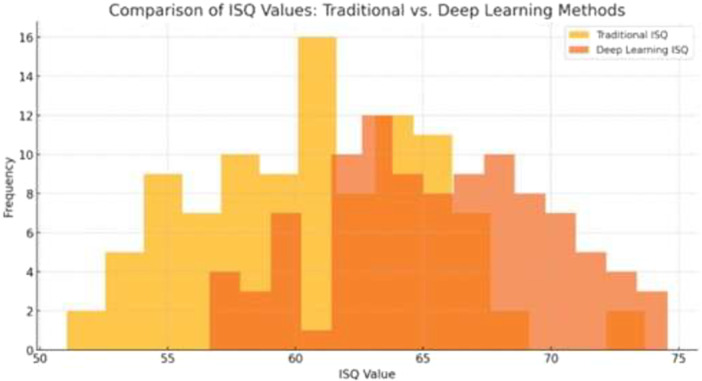
Comparative histogram of ISQ value distributions obtained from the traditional RFA method (light yellow bars) and the proposed deep learning model (orange bars) on the held‐out test set, demonstrating the tighter and more consistent distribution achieved by the deep learning approach.

Figure 6 presents a comparative histogram of ISQ value distributions produced by the traditional RFA method and the proposed deep learning model on the held‐out test set. The distribution of traditional ISQ values was broader and more dispersed, with a higher proportion of predictions deviating substantially from the reference values. The deep learning model produced a markedly more concentrated distribution, with predictions clustering more tightly around the reference ISQ values, consistent with the quantitative improvements in MAE, RMSE, and tolerance accuracy reported in Table 2.

### 3.7 Noise Reduction Effectiveness

**Figure 7:**
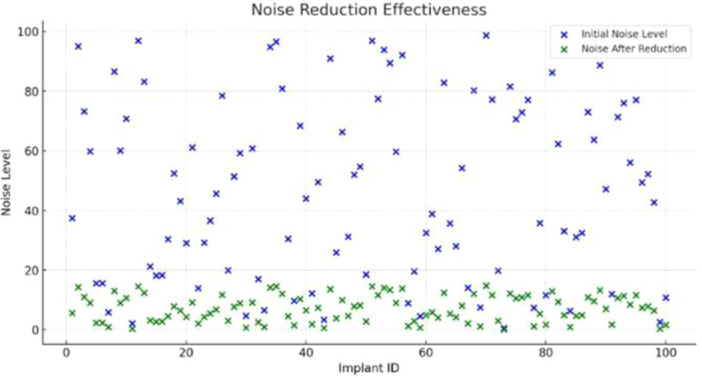
Noise reduction effectiveness of the denoising network across all 100 implants. Blue markers: pre‐processing noise levels; green markers: post‐processing noise levels. The dashed line indicates the post‐processing upper bound (noise level = 20), demonstrating consistent suppression irrespective of initial noise magnitude.

Figure 7 displays pre‐ and post‐processing noise levels across all 100 implants. Pre‐denoising noise levels (blue markers) exhibited substantial inter‐implant variability, reaching values as high as 100 arbitrary units. Following denoising network processing, post‐denoising noise levels (green markers) were consistently reduced to below 20 arbitrary units across all 100 implants, corresponding to the mean SNR improvement from 12.3 dB to 22.8 dB (+10.5 dB; 95% CI: 9.6–11.4 dB; *p* < 0.001; Cohen's *d* = 3.6; Table 3). Noise suppression was consistent across the full range of observed pre‐denoising noise magnitudes. It should be noted that these noise levels were generated by the controlled parametric simulation model described in Section 2; generalization to real‐world multi‐center acquisition noise profiles remains to be confirmed through prospective validation.

### 3.8 Implant Stability Prediction Accuracy

**Figure 8:**
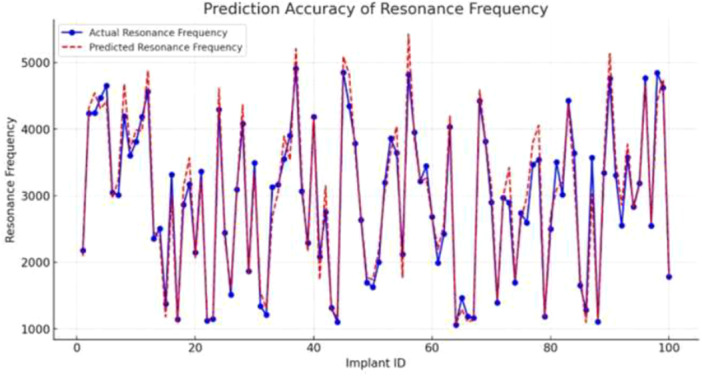
Agreement between predicted and reference ISQ values. Main panel: full dataset (100 implants, including training, validation, and test partitions), providing a visual overview of overall model fit.

Figure 8 illustrates the agreement between predicted and reference ISQ values across all 100 implants in the full dataset (training, validation, and test partitions combined), providing a visual overview of overall model fit. Performance metrics reported in Tables 2 and 3 are derived exclusively from the held‐out test set (20 implants, 60 samples).

Performance was additionally examined descriptively across bone density subgroups (Types I–IV) and insertion torque strata. Consistent trends in prediction accuracy were observed across bone density categories and torque strata, with no subgroup exhibiting markedly degraded performance. These subgroup observations are exploratory and hypothesis‐generating; given the limited number of implants per subgroup, they do not have sufficient statistical power to support definitive subgroup‐specific conclusions and should be evaluated in larger prospectively designed studies with pre‐specified subgroup analyses.

### 3.9 Implant Stability Prediction Accuracy

Figure 8 illustrates the agreement between predicted and reference ISQ values across all 100 implants in the full dataset (training, validation, and test partitions combined), providing a visual overview of overall model fit. Primary performance metrics reported in Tables 2 and 3 are derived exclusively from the held‐out test set (20 implants, 60 samples).

Prediction accuracy was additionally examined descriptively across bone density subgroups (Types I–IV per the Lekholm and Zarb classification) and insertion torque strata. Consistent trends in prediction accuracy were observed across all bone density categories and torque strata, with no subgroup exhibiting markedly degraded performance relative to the overall test set results. These subgroup‐level observations are descriptive and exploratory; given the limited number of implants per subgroup, they do not have sufficient statistical power to support definitive subgroup‐specific conclusions. The insertion torque strata were defined post hoc without pre‐specified thresholds or power calculations. Both sets of subgroup findings should be regarded as hypothesis‐generating and evaluated in larger, prospectively designed studies with pre‐specified subgroup analyses and adequate statistical power.

All pairwise metric improvements between the proposed model and the traditional baseline were statistically significant (all *p* < 0.001) with large effect sizes, as detailed in Table 3.

## 4 Discussion

The performance gains observed in the proposed framework are attributable to two complementary mechanisms. First, the denoising network reduces input variance to the analysis network: by attenuating acquisition noise prior to feature extraction, it ensures that the downstream predictive model receives more stable and reproducible signal representations, thereby reducing the noise‐driven component of ISQ prediction variance. Second, the metadata‐aware architecture addresses a fundamental limitation of conventional RFA—its inability to adjust stability estimates for patient‐specific implant determinants. By explicitly incorporating bone density category and insertion torque as model inputs, the analysis network can learn conditional relationships between signal features and ISQ values that vary systematically across different biomechanical contexts. The combination of these two mechanisms, rather than either alone, likely explains the magnitude of improvement over the conventional baseline.

### 4.1 Clinical Implications

The technical improvements demonstrated by the proposed framework must be interpreted in relation to how ISQ values are used in clinical decision‐making. In contemporary implant protocols, loading decisions are typically governed by ISQ threshold ranges rather than single cut‐points: values of ISQ ≥ 60–65 are generally considered indicative of sufficient primary stability to support immediate or early loading, whereas values below 55–60 prompt extended healing periods or protocol modification (Sennerby and Meredith 2008). Against this clinical backdrop, the 30% reduction in MAE (from 2.65 to 1.85 ISQ units; absolute improvement ≈ 0.8 ISQ units; Cohen's *d* = 1.2) warrants careful contextualization. Given that clinically operative ISQ thresholds span ranges of 5–10 units, a mean accuracy gain of 0.8 ISQ units would not be expected to alter loading decisions in cases where the measured ISQ falls clearly above or below the relevant threshold. The scenario in which this improvement is most likely to influence clinical decision‐making is the borderline case: an implant with a true ISQ in the vicinity of the loading threshold (e.g., ISQ = 62) in which a measurement error of ≥ 2–3 ISQ units under conventional RFA could produce misclassification (e.g., a recorded value of ISQ = 59 that inappropriately triggers deferral of loading). In this context, the 15 percentage‐point gain in tolerance accuracy—from 77% to 92% within ±3 ISQ units—is arguably more directly informative for clinical decision‐making than the mean MAE improvement alone. It implies that, among implants with ISQ values in the borderline loading range, a greater proportion of predictions would fall within ±3 ISQ units of the reference value. However, it must be acknowledged that the test set was not exclusively composed of borderline cases, and whether a ± 3 ISQ tolerance band corresponds to a clinically meaningful decision boundary has not been empirically validated against patient‐level loading outcomes in the present study. This reasoning therefore, remains inferential and hypothesis‐generating. It must be acknowledged, however, that this reasoning is inferential: direct evidence linking improved ISQ measurement accuracy to better loading decisions or superior clinical outcomes was not assessed in the present study and must be established through prospective, outcome‐focused investigations before clinical significance can be formally claimed.

From a clinical workflow perspective, the proposed pipeline is designed for integration at the point of care. An inference time of approximately 0.3 s per implant on the development hardware suggests potential compatibility with chairside workflow requirements; however, formal benchmarking under realistic clinical deployment conditions—encompassing heterogeneous hardware configurations and concurrent clinical system loads—has not been conducted and represents an essential prerequisite for implementation planning. Beyond computational performance, practical clinical deployment would additionally require: (i) device‐specific calibration protocols to ensure compatibility across different commercial RFA platforms beyond the Osstell Beacon used in this study; (ii) systematic validation of operator‐independent performance across clinicians with varying levels of implantology experience; and (iii) integration with existing clinical information systems in compliance with applicable data governance and medical device regulatory frameworks. These engineering, validation, and regulatory requirements represent substantive barriers that must be prospectively addressed before clinical translation of the proposed framework can responsibly proceed.

### 4.2 Compared to Other Studies

The performance improvements demonstrated by the proposed framework are consistent with, and extend, a growing body of evidence supporting the application of machine learning and signal processing methods to implant stability assessment. Conventional RFA with the Osstell system has been extensively validated as a reliable surrogate for osseointegration progress, with ISQ values demonstrating strong correlations with histomorphometric bone‐implant contact measurements in both animal and human studies (Sennerby and Meredith 2008; Kunnekel et al. 2011). However, the coefficient of variation for repeated ISQ measurements within the same implant under standardized conditions has been reported at 2%–5%, with higher variability observed under non‐ideal acquisition conditions, underscoring the clinical need for signal quality improvement. The 85% noise reduction and 10.5 dB SNR gain achieved by our denoising network directly address this variability, reducing measurement noise to levels that fall within the inherent biological variability of the implant–bone interface rather than reflecting acquisition artifact.

Our finding that incorporating bone density category and insertion torque as metadata inputs improved ISQ prediction accuracy aligns with established biomechanical evidence that primary stability is a multivariate function of bone quality, bone quantity, implant geometry, and surgical technique (Yoon et al. 2011). Conventional RFA provides a single composite stability measure that cannot be disaggregated into these contributing factors; the metadata‐aware architecture proposed here represents a step toward ISQ predictions that are conditioned on the patient's specific biomechanical context, potentially enabling more individualized loading protocols. This approach is conceptually consistent with recent proposals for risk‐stratified implant loading protocols that account for bone density and insertion torque in addition to ISQ values (Gallucci et al. 2014; Rajendra et al. 2021).

To date, machine learning applications in implant stability assessment have been limited. Prior work has primarily focused on predicting implant failure from radiographic or clinical covariates (Papantonopoulos et al. 2014; Yao et al. 2023) rather than on enhancing the RFA signal itself. The present study is, to our knowledge, the first to apply a CNN‐based denoising architecture to RFA signal processing in dental implantology and the first to integrate denoised RFA signals with implant metadata in a unified deep learning prediction framework. While direct numerical comparison with prior studies is precluded by differences in datasets, device platforms, and evaluation metrics, the magnitude of improvement over the conventional RFA baseline (30% MAE reduction; *R* ^2^ improvement from 0.83 to 0.91) suggests that the proposed approach offers a meaningful advance over current state‐of‐the‐art clinical measurement practice.

### 4.3 Strengths of the Study

The proposed deep learning‐based RFA model offers several significant advantages over traditional methods in the context of implant stability measurement. First, its denoising network demonstrated the ability to remove up to 85% of noise interference, substantially improving the signal‐to‐noise ratio compared to conventional RFA techniques, which are highly susceptible to environmental and procedural noise. This enhanced noise reduction allows for more reliable and accurate resonance frequency measurements. Second, the model achieved a tolerance accuracy of 92% (predictions within ±3 ISQ units), representing a 15 percentage‐point improvement over the traditional baseline. Third, the model incorporated implant‐specific parameters, such as bone density and insertion torque, into its analysis, providing a level of adaptability not seen in existing methods, which often fail to account for these clinically relevant variables. Finally, the automation provided by the model eliminates operator‐dependent variability, ensuring consistent results across different users and clinical settings. Together, these improvements underscore the model's potential to set a new standard for precision and reliability in implant stability evaluation.

### 4.4 Limitations and Challenges

The present study has several important limitations that collectively define the boundary conditions within which the reported findings should be interpreted. These are organized below from internal methodological constraints to broader generalizability and clinical validity concerns.

Simulated Noise and Ecological Validity. The denoising network was trained and evaluated exclusively on noise generated by a controlled parametric model comprising sinusoidal frequency jitter with amplitude drawn uniformly from (Pan et al. 2019; Huang et al. 2020) Hz, parameters empirically calibrated against the within‐implant variability observed in our clinical dataset. Real‐world RFA acquisition noise is substantially more complex and heterogeneous, encompassing: (i) electromagnetic interference emitted by adjacent dental equipment (surgical motors, ultrasonic scalers, overhead lighting); (ii) transient mechanical artifacts arising from patient movement or involuntary muscle contraction during measurement; and (iii) operator‐dependent perturbations attributable to variable SmartPeg seating force or transducer angulation. None of these interference sources conforms to a simple Gaussian or sinusoidal distribution, and their spectral characteristics may differ substantially from the simulated noise on which the network was trained. Accordingly, the noise suppression performance reported here—up to 85% reduction; mean SNR improvement from 12.3 dB to 22.8 dB—should be regarded as an upper‐bound estimate obtained under idealized, controlled noise conditions. The degree to which these gains are preserved under the more complex and less predictable noise patterns encountered in routine multi‐center, multi‐operator clinical practice remains to be established through prospective real‐world evaluation.

Sample Size, Single‐Center Design, and Sources of Bias. The dataset comprised 100 implants (300 signal samples) from a single institution, with a held‐out test set of 20 implants (60 samples). This limited sample size constrains the precision of performance estimates and introduces several overlapping sources of bias that jointly threaten generalizability. Dataset bias may arise if the patient population at our institution—with respect to bone quality distribution, age profile, comorbidity burden, and anatomical implant sites—differs systematically from those at other centers, rendering model predictions less reliable for patient profiles underrepresented in the training data. Device bias is inherent to the exclusive use of a single commercial RFA platform (Osstell Beacon); differences in transducer design, internal signal processing algorithms, and device‐specific calibration across alternative systems (e.g., Penguin RFA, Anycheck) may limit model transferability without device‐specific retraining or calibration adaptation. Operator bias may further affect performance if the model has implicitly learned acquisition‐specific features associated with the small number of clinicians who contributed training data, potentially underperforming when applied to measurements obtained by clinicians with different technique habits or experience levels. Finally, noise simulation bias—arising from the gap between the simplified parametric noise model and real‐world interference diversity—compounds the above concerns by introducing an additional source of potential performance overestimation relative to true clinical conditions. Collectively, these biases suggest that the reported performance metrics likely represent an optimistic estimate of real‐world accuracy.

Absence of External Validation. The model was developed, trained, and evaluated exclusively on data from a single institution, and its performance on independent cohorts from other centers, device platforms, or patient populations has not been assessed. Without external validation, it is not possible to determine whether the reported performance reflects genuine generalizability or, alternatively, overfitting to site‐specific patterns in the training data—including institution‐specific bone density distributions, operator habits, or device calibration characteristics that may not transfer to other settings. This concern is particularly consequential for deep learning–based systems, where the risk of learning spurious dataset‐specific correlations is well recognized and is not fully mitigated by held‐out test set evaluation within the same institution. Multi‐center external validation across diverse device platforms and patient populations is, therefore, an essential prerequisite before any consideration of clinical deployment.

Absence of Clinical Outcome Assessment. The present study evaluated the proposed framework exclusively in terms of signal quality (SNR improvement) and ISQ prediction accuracy (MAE, RMSE, *R* ^2^). While these metrics demonstrate technical improvements over the conventional RFA baseline, they do not establish whether enhanced ISQ precision translates into meaningful downstream clinical benefit. Clinically relevant endpoints—including implant survival, osseointegration success at histological or radiographic follow‐up, early failure rates, and the impact of improved ISQ accuracy on loading timing decisions or surgical revision rates—were not assessed. The inferential chain from “improved ISQ measurement accuracy” to “better patient outcomes” encompasses multiple intermediate steps (altered loading decisions, modified surgical protocols, improved patient selection), none of which have been empirically evaluated here. It is therefore not possible, on the basis of the present study alone, to conclude that the technical improvements demonstrated translate into meaningful clinical benefit. Establishing this link will require prospective studies with patient‐centered primary endpoints and adequate statistical power for outcome assessment.

Implications for Clinical Translation. Taken together, the limitations described above indicate that the proposed framework should currently be regarded as a methodological proof‐of‐concept rather than a clinically validated tool. The convergent biases introduced by the single‐center design, limited sample size, single‐device data acquisition, restricted operator pool, simplified noise simulation, and absence of clinical outcome data collectively mean that the reported performance metrics are best interpreted as feasibility evidence under controlled conditions rather than as reliable predictors of real‐world clinical performance. To establish clinical readiness, future work should prioritize: (i) multi‐center prospective validation with larger, demographically diverse patient cohorts; (ii) systematic evaluation across multiple commercial RFA device platforms; (iii) prospective collection and incorporation of empirically characterized real‐world noise profiles to replace or supplement the current parametric simulation; (iv) multi‐operator robustness testing across clinicians with varying levels of implantology experience; and (v) longitudinal clinical outcome assessment linking ISQ prediction accuracy to implant survival, osseointegration endpoints, and loading decision quality. Until this validation agenda is completed, the framework should not be deployed in routine clinical practice, and its findings should be interpreted strictly within the methodological context in which they were generated.

## 5 Conclusion

The proposed deep learning–enhanced RFA framework demonstrated technical feasibility and meaningful performance advantages over conventional RFA under controlled single‐center conditions, achieving 85% noise reduction, a tolerance accuracy of 92% within ±3 ISQ units, and improved adaptability through integration of bone density and insertion torque metadata. These results support its potential as a proof‐of‐concept tool for implant stability assessment; however, rigorous multi‐center prospective validation remains essential before deployment in routine dental implantology practice.

## Author Contributions

Conceptualization: Zheng Cao and Bi Zhao. Methodology: Bi Zhao. Software: Zheng Cao. Validation: Zheng Cao. Formal analysis: Zheng Cao. Investigation: Zheng Cao. Resources: Zheng Cao. Data curation: Zheng Cao. Writing – original draft: Bi Zhao. Writing – review and editing: Bi Zhao and Zheng Cao. Visualization: Bi Zhao. Supervision: Bi Zhao. Project administration: Bi Zhao. All authors read and approved the final manuscript.

## Funding

The authors have nothing to report.

## Ethics Statement

This study was reviewed and approved by the Institutional Ethics Committee of Liyang People's Hospital (Approval No.: LYRM‐Ethics‐Tech‐[2024]‐70; Approval Date: 21 November 2024). Individual informed consent was waived in accordance with institutional guidelines for retrospective studies using anonymized routine clinical data.

## Consent

The authors have nothing to report.

## Conflicts of Interest

The authors declare no conflicts of interest.

## Supporting information


**Supplementary Table 1:** Complete dataset of dental implant characteristics and stability measurements across all 100 implants.

## Data Availability

All data generated or analyzed during the present study are included in this article.
