# Supplementary material for: Deep Learning–Enhanced Resonance Frequency Analysis for Dental Implant Stability Assessment
Source: Clin Exp Dent Res. 2026 Mar 31;12(2):e70342. doi: 10.1002/cre2.70342 (PMC13140712; doi:10.1002/cre2.70342)
Supplement: Supplementary file 1 — Supplementary Table 1: Complete dataset of dental implant characteristics and stability measurements across all 100 implants. [file CRE2-12-e70342-s001.pdf]

Supplementary Table 1. Complete Dataset of Dental Implant Characteristics and Stability Measurements Across All 100 Implants.

Implant_ID	Bone_Density	Insertion_Torque (Ncm)	Traditional_ISQ	Deep_Learning_ISQ	Noise_Level
1	High	27.95	46.36	67.20	Low
2	Low	20.22	58.17	59.92	Medium
3	High	52.62	63.25	74.56	High
4	High	48.27	53.89	72.37	Medium
5	Low	49.16	62.68	70.45	Low
6	Low	50.85	55.43	68.32	Medium
7	High	22.96	63.10	65.06	Low
8	Medium	34.34	59.20	66.77	Medium
9	High	24.63	58.06	69.19	High
10	High	54.52	55.57	62.49	Medium
11	High	44.93	58.22	63.22	Medium
12	High	33.24	62.78	58.32	Low
13	Low	22.54	65.22	61.91	Low
14	High	32.44	62.63	71.65	Low
15	Medium	33.01	66.82	63.66	Medium
16	Low	49.18	72.70	63.73	High
17	Medium	45.50	58.38	66.27	Low
18	Medium	55.49	58.97	58.55	Low
19	Medium	38.89	52.80	68.78	Medium
20	Medium	24.78	65.95	70.07	Low
21	Low	48.53	66.50	66.51	High
22	Low	50.43	55.66	71.61	High
23	Medium	42.45	63.09	59.69	Low
24	Medium	50.84	66.09	72.37	Low
25	Low	39.75	61.13	62.08	Medium
26	Low	40.91	64.24	59.88	High
27	Low	37.10	60.87	64.05	High
28	High	21.02	53.92	72.49	Low
29	High	24.32	65.25	64.92	Medium
30	High	21.26	66.63	61.27	High
31	Medium	45.46	63.67	59.82	High
32	High	32.57	55.23	64.58	Medium
33	Medium	40.34	56.24	57.02	Medium
34	Medium	56.30	54.35	59.80	High
35	High	29.97	63.85	71.17	High
36	Medium	36.42	66.34	70.29	Medium
37	High	50.22	62.12	64.49	High

Implant_ID	Bone_Density	Insertion_Torque (Ncm)	Traditional_ISQ	Deep_Learning_ISQ	Noise_Level
38	High	29.15	64.70	63.14	Low
39	Low	23.08	55.66	65.23	Low
40	High	31.59	60.73	74.39	Medium
41	Low	26.45	53.15	66.62	Medium
42	High	57.19	56.14	68.64	Medium
43	High	52.32	64.39	64.19	Low
44	Low	45.34	58.80	65.24	High
45	Low	54.86	66.05	67.59	Low
46	High	52.15	62.69	65.50	High
47	Medium	27.46	73.67	57.41	Low
48	Low	55.70	60.47	70.12	High
49	Medium	41.57	52.97	62.54	Low
50	Medium	52.30	59.83	73.45	Medium
51	Medium	55.84	55.18	67.75	Low
52	Low	32.72	64.89	65.28	High
53	Medium	24.40	60.21	66.76	Low
54	Low	29.12	59.31	64.57	Low
55	Medium	37.08	59.38	63.05	Low
56	High	52.72	63.70	65.83	Medium
57	High	54.43	57.74	68.54	Low
58	Low	20.28	63.89	63.14	Medium
59	High	40.43	65.23	62.51	Medium
60	High	36.70	58.29	63.58	Low
61	Medium	28.88	55.37	68.81	Low
62	Low	24.79	57.44	70.81	Low
63	Medium	33.50	63.55	66.45	High
64	Medium	57.72	60.46	67.33	Low
65	Medium	32.93	63.15	62.48	Medium
66	Medium	40.75	68.81	68.51	Low
67	Medium	48.12	61.15	67.21	High
68	Medium	34.55	55.96	62.16	Low
69	Medium	58.87	65.29	57.85	High
70	Low	58.50	60.26	60.12	High
71	High	30.07	64.36	62.32	Low
72	Medium	39.89	65.33	69.65	Medium
73	Medium	32.04	55.20	71.21	High
74	Medium	31.39	66.91	63.24	Low
75	Medium	21.48	64.53	63.37	Low

Implant_ID	Bone_Density	Insertion_Torque (Ncm)	Traditional_ISQ	Deep_Learning_ISQ	Noise_Level
76	Medium	44.38	56.98	66.05	Medium
77	Medium	40.11	61.52	65.05	High
78	High	22.06	61.29	68.72	High
79	High	31.15	60.12	62.91	Low
80	Medium	56.33	64.36	68.80	Low
81	High	29.58	67.19	64.04	Medium
82	Low	25.80	60.04	68.42	Medium
83	Medium	39.58	66.65	63.92	High
84	Low	59.43	64.94	62.55	High
85	Low	29.68	61.16	72.65	Low
86	Medium	46.89	60.88	69.26	High
87	High	50.46	54.24	57.83	Medium
88	Low	29.51	52.50	63.21	Medium
89	Medium	49.13	60.83	69.82	Low
90	Low	34.71	55.72	60.21	Medium
91	Low	45.29	59.80	68.08	Medium
92	Low	45.34	57.33	67.67	Medium
93	Low	41.43	51.06	62.20	Medium
94	High	23.61	61.79	64.96	Low
95	Low	53.41	57.93	56.64	Low
96	Low	32.83	60.40	72.08	High
97	Low	27.46	55.53	63.76	Medium
98	High	21.63	58.33	68.22	High
99	Low	43.64	67.80	67.46	Low
100	Low	47.10	60.64	70.43	Low
